# Axonal regeneration in zebrafish spinal cord

**DOI:** 10.1002/reg2.99

**Published:** 2018-04-22

**Authors:** Sukla Ghosh, Subhra Prakash Hui

**Affiliations:** ^1^ Department of Biophysics Molecular Biology and Bioinformatics University of Calcutta 92 A. P. C. Road Kolkata 700009 India; ^2^ Victor Chang Cardiac Research Institute Lowy Packer Building, 405 Liverpool St Darlinghurst NSW 2010 Australia.

**Keywords:** axonal regeneration, central nervous system, peripheral nervous system, spinal cord injury (SCI), zebrafish

## Abstract

In the present review we discuss two interrelated events—axonal damage and repair—known to occur after spinal cord injury (SCI) in the zebrafish. Adult zebrafish are capable of regenerating axonal tracts and can restore full functionality after SCI. Unlike fish, axon regeneration in the adult mammalian central nervous system is extremely limited. As a consequence of an injury there is very little repair of disengaged axons and therefore functional deficit persists after SCI in adult mammals. In contrast, peripheral nervous system axons readily regenerate following injury and hence allow functional recovery both in mammals and fish. A better mechanistic understanding of these three scenarios could provide a more comprehensive insight into the success or failure of axonal regeneration after SCI. This review summarizes the present understanding of the cellular and molecular basis of axonal regeneration, in both the peripheral nervous system and the central nervous system, and large scale gene expression analysis is used to focus on different events during regeneration. The discovery and identification of genes involved in zebrafish spinal cord regeneration and subsequent functional experimentation will provide more insight into the endogenous mechanism of myelination and remyelination. Furthermore, precise knowledge of the mechanism underlying the extraordinary axonal regeneration process in zebrafish will also allow us to unravel the potential therapeutic strategies to be implemented for enhancing regrowth and remyelination of axons in mammals.

## 1 INTRODUCTION

Among the vertebrates, teleost fish and urodele amphibians have a remarkable capacity to regenerate their axons after spinal cord injury (SCI) (Becker & Becker, 2014; Clarke, Alexander, & Holder, 1988; Hui, Dutta, & Ghosh, 2010; Hui, Monaghan, Voss, & Ghosh, 2013). In contrast to mammals, zebrafish serve as an important model because two efficient programs of neural regeneration exist in adult fish cord, namely neurite outgrowth and remyelination. The extent of axonal regeneration has been evaluated by using various injury paradigms, such as transection or crush injury in zebrafish spinal cord (Becker, Wulliman, Becker, Bernhardt, & Schrachner, 1997; Hui et al., 2010). However, the lack of long distance axonal regeneration in the mammalian central nervous system (CNS) has been ascribed to inadequate capability of intrinsic growth of neurons and the creation of extrinsic inhibitory milieu after injury (Huebner & Strittmatter, 2009). The regenerative ability of CNS axons differs between mammals and fish, although some of the molecular and cellular pathways underlying axonal regeneration are similar. The remarkable differences in regenerative capacity between the CNS and peripheral nervous system (PNS) are due to differences in the intrinsic capabilities of the injured neurons and differential environmental cues. Thus, in this review, we intend to dissect out the similarities and differences in the cellular and molecular mechanisms that regulate axonal regeneration between the CNS and PNS in both fish and mammals since further insight could be pivotal for inducing successful axonal repair and targeting innervations in higher organisms.

It is important to take into consideration that zebrafish represent a powerful experimental model for studying many neurogenetic disorders because the zebrafish genome has been sequenced and annotated and most zebrafish genes are highly conserved in mammals with a zebrafish ortholog identified for 70% of human genes (Howe et al., 2013). Comparative analysis of neuroanatomy between zebrafish and human central and peripheral nervous systems revealed a similar structural organization, including cell types and neurons. Thus by taking advantage of new tools available for genetic manipulation such as genome editing, high‐throughput DNA/RNA sequencing, in vivo imaging, etc. most of human physiology and pathologies can be modeled in zebrafish (Babin, Goizet, & Raldua, 2014; Howe et al., 2013; Kabashi, Brustein, Champagne, & Drapeau, 2011). As some common molecular mechanisms after SCI such as microglial/macrophage response and faithful recapitulation of the myelinating program exist during regeneration and genes identified in the fish are conserved in higher vertebrates (D'Rozario, Monk, & Petersen, 2017), zebrafish offer a promising tool for translational research.

## 2 AXONAL INJURY RESPONSE IN MAMMALIAN CNS AND PNS

Adult mammalian CNS axons demonstrate very little capacity to regenerate injured axons. The initial injury impact destroys many neurons, glia, and endothelial and meningeal cells, and the loss is further intensified by subsequent secondary degenerative response. The loss of astrocytes leads to abnormal ionic homeostasis whereas oligodendrocyte loss contributes to poor myelination and impaired axonal activity (Grossman, Rosenberg, & Wrathall, 2001). Following an injury the distal part of severed axons, that had lost contact with the neuronal cell bodies, degenerates. Occasionally the proximal segments survive and grow short sprouts but fail to regenerate and re‐innervate appropriate targets (Ramon & Cajal, 1928; Thuret, Moon, & Gage, 2006), despite the attempt of the injured axons to navigate through the lesion environment. Upon injury, axons exhibit dystrophic growth cones identified as sterile clubs by Ramon and Cajal (1928) and later confirmed by others (Ertürk, Hellal, Enes, & Bradke, 2007; Tom, Steinmetz, Miller, Doller, & Silver, 2004). The formation of dystrophic end balls at the ends of lesioned axons is believed to be the reason for the inability for axonal regrowth. Morphologically these swollen entities contain disrupted cytoskeleton and accumulated organelles that persist for months to years after SCI (Hill, 2017; Hill, Beattie, & Bresnahan, 2001; Ruschel et al., 2015). Injured axons of the PNS can generate a new motile growth cone within hours of injury whereas injured CNS axons retract and form a retraction bulb. The formation of motile‐growth‐cone‐like structures also refers to a key difference between regeneration competent and incompetent axons. Further advancement of our understanding of retraction bulb formation compared to growth cone formation and growth cone collapse at different stages after injury is pivotal to recognizing the outcome and complexity of axonal injury response.

The landmark experiments by Aguayo, David and Bray (1981) showed that some injured CNS axons retain a limited capacity for regrowth and can regrow over a long distance in a permissive environment of sciatic nerve graft but cannot reintegrate into the CNS. Moreover, sprouting or regeneration without establishment of actual synaptic target innervations does not have any functional significance. Later, others showed that myelin from peripheral nerve is growth permissive whereas myelin from the CNS strongly inhibits nerve growth (Caroni & Schwab, 1988). It is also known that adult PNS neurons retain regenerative ability after injury and both sensory and motor axons can regenerate over a long distance. Thus substantial anatomical regeneration leads to functional recovery (Abe & Cavalli, 2008). Dorsal root ganglia (DRG) are unique as their axons bifurcate to innervate peripheral targets like skin and muscles whereas the central branch supplies sensory information to the CNS. While the peripheral branch of DRG can regenerate following injury, the central branches are unable to do the same. Thus, the CNS and PNS respond differently to injury. This striking difference in regenerative ability is due to intrinsic properties of injured PNS and CNS neurons as well as to the differential extracellular environment of PNS and CNS axons. The difference in response to injury also resides in the glial population which may elicit either a pro‐ or anti‐regenerative response (Lutz & Barres, 2014). A deeper understanding of these cellular and molecular mechanisms highlights the differential ability of CNS and PNS axons and would allow us to devise future therapeutic strategies to induce effective axonal regeneration in the CNS.

## 3 AXONAL INJURY IN FISH CNS LEADS TO FUNCTIONAL RECOVERY

**Figure 1:**
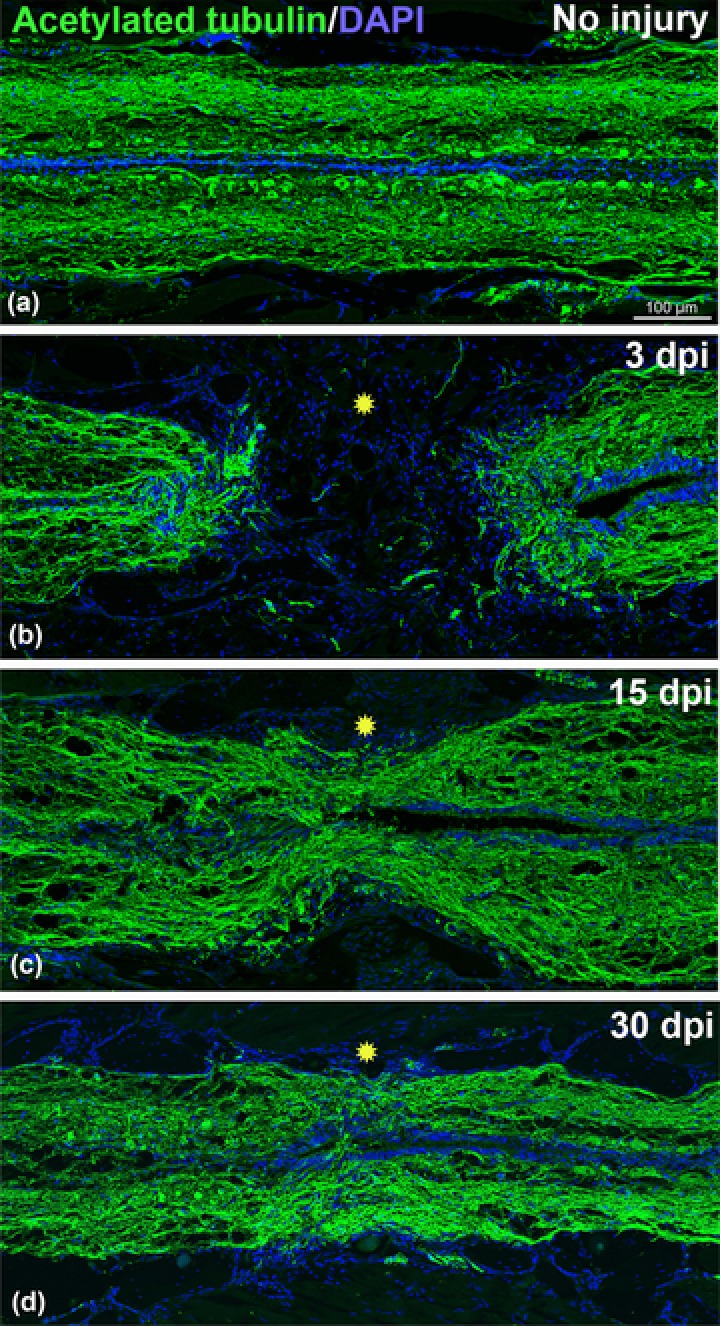
Time course of axonal regeneration in adult zebrafish stained with acetylated‐tubulin (green) and DAPI (blue). (A) Uninjured adult spinal cord. (B) 3‐day post transected spinal cord showing complete loss of axonal connections (green) in the injury epicenter (yellow star). (C) 15‐day post transected spinal cord showing some regenerated axons passing through the injury epicenter (yellow star). (D) A 30‐day post transected spinal cord showing significant numbers of regenerated axons passing through the injury epicenter (yellow star). Significant axonal regrowth can be observed compared to uninjured cord. All the images are of the same magnification. Scale bar 100 μm (A, B, C, and D)

Adult zebrafish have the capability of robust axonal regeneration following spinal cord and optic nerve injuries. Severed axons can regrow following injury and as a consequence functional restoration can take place, which is quite remarkable. After injury, loss of function reflects the level of injury, i.e., fishes are paralyzed caudal to the lesion site but regain their normal swimming behavior within 4−6 weeks (Figure 1) (Becker et al., 2004; Dias, Yang, Ogai, Becker, & Becker, 2012; Hui et al., 2010; van Raamsdonk, Maslam, de Jong, Smit‐Onei, & Velzing, 1998). Regeneration of some axons after SCI is robust, e.g., severed axons of brainstem neurons can project across a significant distance—approximately 3.5 mm beyond the lesion site (Becker et al., 1997). But for others, axonal regrowth to a large extent is variable, e.g., Mauthner neurons exhibit poor axonal regrowth, although it can be augmented by changing intracellular signaling by adenosine 3′,5′‐cyclic monophosphate (cAMP) (Bhatt, Otto, Depoister, & Fetcho, 2004). Similarly, dorsal root axons and ascending axons of intraspinal neurons do not show any significant regrowth (Becker, Leiberoth, Becker, & Schachner, 2005; Becker et al., 1998), whereas descending mono‐aminergic axons are capable of axonal regrowth for only a few micrometers into the distal stump. However, the volume of white matter renewal is not 100% compared to the original uninjured cord (Hui et al., 2010) and most of the regenerating axons extend through gray matter rather than through white matter, perhaps taking support of macrophages and microglia (Becker & Becker, 2001).

## 4 NEUROTRAUMA AND INFLAMMATORY RESPONSE

Traumatic injury to the nervous system triggers an inflammatory reaction. Inflammation in the CNS and PNS differs from each other. Based on various studies we compared the inflammatory response between mammalian and fish SCI highlighting how it differs in terms of magnitude and involvement of different cell types along with the effector molecules.

### 4.1 Microglia/macrophage mediated response after mammalian SCI

The inflammatory response after CNS injury is primarily due to activation of microglia and recruitment of peripherally derived macrophages which are known to contribute to secondary degenerative response within the mammalian CNS (Fitch & Silver, 2008). Both these cells respond to injury in different proportions depending on the type and extent of injury and may produce various cytotoxic to trophic factors which can play a deleterious as well as a beneficial role in CNS tissue. Resident CNS cells upregulate pro‐inflammatory cytokines and chemokines within the first hour after injury which may cause neuronal death and destruction in the vicinity of the injury and thus may play a crucial role in disconnecting existing neuronal connections (Banati, Gehrmann, Schubert, & Kreuzberg, 1993; Giulian, Chen, Ingeman, George, & Noponen, 1989). Microglia become activated within 1 day after injury and play a role in phagocytosis of tissue debris, thus becoming involved in scavenging, resisting infections, and restoration of tissue homeostasis (Hanisch & Kettenmann, 2007; Jin & Yamashita, 2016). The role of microglia in clearing cell debris is amplified in CNS injury, although their phagocytic capacity may be limited compared to blood‐borne macrophages (Neumann, Kotter, & Franklin, 2008). After CNS injury microglia may produce anti‐inflammatory cytokines such as interleukin 4 (IL‐4), IL‐10 and transforming growth factor β (TGF‐β) having a neuroprotective role (Hanisch & Kettenmann, 2007; Jin & Yamashita, 2016; Streit, 2005).

The inflammatory response due to microglial production of cytokines or chemokines recruits peripheral immune cells such as monocytes, neutrophils, dendritic cells, and T‐lymphocytes at the injured site in different phases of injury. Most importantly, in mammalian SCI, hematogenous macrophages and microglia persist indefinitely at the injury site (Donnelly & Popovich, 2008) whereas depletion of macrophages improves recovery and augments reparative macrophage phenotypes increasing axon growth and motor activity (Popovich et al., 1999; Schwartz & Yoles, 2006). Moreover, delayed and limited recruitment of macrophages into the lesion site and the persistent presence of myelin debris create a non‐conducive environment for axon growth leading to CNS regeneration failure (George & Griffin, 1994; Perry, Brown, & Gordon, 1987; Schwab, Kapfhammer, & Bandtlow, 1993).

Macrophages phagocytose debris and secrete neurotrophic factors and play a direct role in axonal retractions and axonal dieback which occur extensively following injury. Microglial response after injury also contributes to scar formation (Dibaj et al., 2010; Silver, Schwab, & Popovich, 2015). Macrophages engulf axonal fragments at the vicinity of the injured site where inflammation is most intense (Gensel et al., 2009). Activated microglia/macrophages release matrix metalloproteinase‐9 (MMP‐9) and are implicated in blood−brain barrier disruption, neutrophil invasion, and secondary myelin degradation. Blood derived macrophages rather than resident microglia are responsible for prolonged dieback of injured axons (Evans et al., 2014), which is initiated by MMP activity (Horn, Busch, Hawthorne, Van Rooijen, & Silver, 2008). Acute, transient upregulation of MMP‐9 and delayed but persistent upregulation of MMP‐2 has been reported in SCI and spinal nerve injury (Noble, Donovan, Igarashi, Goussev, & Werb, 2002; Verslegers, Lemmens, Van Hove, & Moons, 2013). MMP‐9 and MMP‐2, in particular, are predominantly recognized as key players in clearing the path for axons to regrow by breaking chondroitin sulfate proteoglycan (CSPG) scar tissue. An in vitro study also revealed that activated macrophages cause marked axonal retraction which can be inhibited by functionally blocking MMP‐9, but not MMP‐2 (Busch, Horn, Silver, & Silver, 2009).

Macrophages are exposed to the milieu of injured CNS and differentiate into a functionally distinct subset of cells with differential effect on neuronal survival and axonal regrowth (Kigerl et al., 2009). M1 type macrophages responsible for interferon‐γ (IFN‐γ) and Toll‐like receptor signaling secrete pro‐inflammatory cytokines which augment inflammation and removal of debris and cause axonal dieback (Horn et al., 2008; Kigerl et al., 2009), whereas M2 type macrophages are alternatively activated, cause cell proliferation and migration, release growth factors such as neurotrophins, enhance oligodendrocyte progenitor cell (OPC) differentiation, remyelination, and axonal regrowth, and reduce axonal dieback. So M2 macrophages initiate anti‐inflammatory responses (Kigerl et al., 2009; Miron et al., 2013) and can overcome axon growth inhibition by CSPG and myelin (Kigerl et al., 2009). Mammalian SCI response begins with an early pro‐inflammatory response. The M1 polarized macrophages persist long after injury exerting neurotoxic effects leading to chronic inflammation and impaired axonal regeneration (Beck et al., 2010; Kigerl et al., 2009).

### 4.2 Macrophages/microglia, after zebrafish SCI, exhibit controlled inflammatory response and augment rapid removal of myelin debris

Several chemokines and cytokines are upregulated after SCI in zebrafish (Hui et al., 2014), and among these IL‐4r, interferon‐1 (IFN‐1), and transforming growth factor beta 1 (TGFβ‐1) are also upregulated in mammalian CNS injury. Similar to other vertebrates, zebrafish microglia express typical vertebrate macrophage genes. Furthermore the expression of many transcriptional regulators, immune pathogen receptors, and pruning associated genes, which are also found in mammals, suggests functional conservation between mammals and fish (Oosterhof et al., 2017).

The secretion of MMPs can be seen in both mammalian and fish CNS injury. Dynamic expression of four specific MMPs (MMP‐2, MMP‐9, MMP‐13a, and MMP‐14) during different phases of retinotectal regeneration has been reported in zebrafish (Lemmens et al., 2016; McCurley & Callard, 2010). Our microarray analysis data demonstrate upregulation of several MMP genes in different phases after SCI in adult zebrafish. In the absence of glial scar in zebrafish CNS injury, we do not see upregulation of MMP2. Most importantly, both MMP‐9 and MMP‐13 are commonly expressed after SCI and optic nerve crush injury, probably indicating their implication in injury response and axonal regrowth (Table 1) (Hui et al., 2014; McCurley & Callard, 2010).

**Table 1:** Summary of events and related molecules involved in CNS and PNS regeneration

Events after injury	Mammalian SCI	Zebrafish SCI	PNS regeneration
Cell death	Glial death Neuronal death and DAMPs (IL‐1α, IL‐33, HMGB1, S‐100β) (Gadani, Walsh, Lukens, & Kipnis, 2015)	HMGB1 (Fang et al., 2014)	IL‐33 (Gadani et al., 2015)
Inflammatory response Microglia/macrophage activation and migration Cytokine/chemokines MMPs M1/M2 macrophage gene M1 M2	+++, ATP and P2Y12 (Davalos et al., 2005; Haynes et al., 2006) IL‐1, IL‐8, MCP, IL‐16, TNF‐α CXCL1, 9, 10, and 12 (Knerlich & Held‐Feindt, 2015) MMP‐2, MMP‐9 (Verslegers Lemmens, Van Hove & Moons, 2013) Hydrogen peroxide, MMP, TNF‐α, IL‐1β, iNOs, superoxide, CD18, CD86, CD16/32 (Czeh, Gressens & Kaindl, 2011; Kigerl et al., 2009) IL‐10, arginase 1, CD163, CD14, CD206, neurotrophins	+++, ATP and P2Y12 (Sieger, Moritz, Ziegenhals, Prykhozhij & Peri, 2012) ccl1, ccrl1a, cmklr1, crfb8, cxcl12b, cxcr3.2, il1b, il4r, il22, irf10, irf11, irf8, irf9 (Hui et al., 2014) MMP‐9, MMP‐13 (Hui et al., 2014) nos and caspa (Hui et al., 2014) scarb1, scarb2, il4r, tgfβ1, vegfa, tgifl, arg2 (Hui et al., 2014)	+++, ATP secretion by Schwann cell (Jung, Jo, Kwon & Jeong, 2014) IL‐6, IL‐1α, IL‐1β, IL‐1m, Cxcl2, Ccl7, Cxcl5, Ccr1, Cxcl1, Ccl2, Ccl20, Ccl3, and Ccr5 (Li et al., 2013) IL‐6, LIF, and CNTF (Abe & Cavalli, 2008) IL‐6, IL‐10, TNF (Xing et al., 2017) MMP‐2, MMP‐3, MMP‐9, MMP‐13a, MMP‐14 (Lemmens et al., 2016; Li et al., 2013; McCurley & Callard, 2010) Lbp, Fegr3a, Cxc (Li et al., 2013)
Immune response Leucocyte invasion	Neutrophilic granulocyte	Leucocyte subtypes?	Neutrophil, oncomodulin (Kurimoto et al., 2013)
Glial response Astrogliosis/glial scar CSPG and ECM molecules	+++, proliferation and migration Upregulation of CSPGs, GAG, tenascin‐C, thrombospondin (Silver & Miller, 2004; Silver et al., 2015)	−−−, absence of CSPG proliferation, migration, formation of glial bridge, upregulation of laminin, collagen XII, fibronectin, integrins (Becker & Becker, 2014; Goldshmit et al., 2012; Hui et al., 2010; Wehner et al., 2017)	‐/? Laminin, collagen IV, integrin, HSPGs (Bunge, Clarke, Dean, Eldridge & Bunge, 1990)
Axon guidance	Slit, semaphorin (3A), syn‐CAM, neuroligin, and ephrin B3 (Hollis, 2016; Onishi, Hollis & Zou, 2014)	Tenacin‐R/CSPG, ephrin A5b, A2 (Becker & Becker, 2014) Ephrin B1, ephrin B3 netrin1a, netrin1b, plexina4, robo1, robo2, slit1b, slit3, sema3ab, and sema3h, Wnt PCP pathway (Hui et al., 2014), Wnt/b catenin signaling (Strand et al., 2016; Wehner et al., 2017)	Sema3A, netrin, Ih3, glycosyltransferase (Isaacman‐Beck, Schneider, Franzini‐Armstrong & Granato, 2015; Rosenberg et al., 2012)
Axon growth promotion (RAGs) Myelin associated inhibitors Axon regeneration inhibitors	‐/ ? (Afshari, Kappagantula & Fawcett, 2009; Sun & He, 2010) Stat3 boost CNS axonal regeneration (Bareyre et al., 2011; Mehta, Luo, Park, Bixby & Lemmon, 2016) +++ Nogo‐A, MAG, OMgp myelin lipid sulfatide (Mukhopadhyay, Doherty, Walsh, Crocker & Filbin, 1994; Wang et al., 2002; Yiu & He, 2006) Pten/mTOR Socs3	+++ Tubulin, L1.1, zRICH proteins, flotillins, reggie 1 and 2, KLF 6 and 7, ATF‐3, cAMP, Socs3/STAT3, GAP‐43, FGF (Becker et al., 2004; Goldshmit et al., 2014; Hui et al., 2014; Veldman, Bemben, & Goldman, 2010) +++ Nogo‐A without delta 20 domain, MAG (Abdesselem, Shypitsyna, Solis, Bodrikov & Stuermer, 2009; Shypitsyna et al., 2010) Ptena (Liu, Yu & Schachner, 2014) Socs?	+++ ATF‐3, c‐Jun, HSp27, Sprr1a, GAP‐43, Sox‐11, Socs3/STAT3 (Bareyre et al., 2011; Bonilla, Tanabe & Strittmatter, 2002; Jankowski et al., 2009; Raivich et al., 2004; Seijffer, Allchorne & Woolf, 2006) –– Except MAG (Siglec‐4) (Huebner & Strittmatter, 2009; Lehmann, Gäthje, Kelm & Dietz, 2004) Pten (Ohtake, Hayat & Li, 2015) Socs?
Demyelination and remyelination Demyelination Myelin composition Remyelination	+++, myelin toxic product stays Myelin structural protein PLP/DM20, MBP (Bromsale & Halpern, 2002; Schweitzer et al., 2006) ‐/? OPC	+++, rapid myelin debris clearance by macrophage DM1α, DM2α MBP, Mpz (Bai et al., 2011), claudin‐K (Bromsale & Halpern, 2002; Munzel et al., 2012) +++ OPC?, Schwann cell progenitor (Hui, Nag & Ghosh, 2015)	+++, debris clearance by macrophage/Schwann cell MBP, P_o_ (Mpz) (Bai et al., 2011) Claudin‐K (Schweitzer, Becker, Becker & Schachner, 2003) +++ Schwann cell progenitor GDNF, artemin, CTNF, LIF, BDNF, NGF (Arthur‐Farraj et al., 2012)

‐/?, weak or negative expression; +++, mammalian SCI; +++, Zebrafish SCI; ‐‐, PNS regeneration (negative expression).

The spatiotemporal activation and distribution of microglia and macrophages after SCI in zebrafish differ from mammals and we observe early activation of microglia and infiltration of blood‐borne macrophages (2−3 days post injury) in the wound site (Hui et al., 2010). In mammals macrophages persist at the injury site for a long time after SCI (42 days post injury in rodents, 12 months post injury in humans) (Fleming et al., 2006; Kigerl, McGaughy, & Popovich, 2006), whereas in adult zebrafish cord depletion of blood‐borne macrophages was observed 10 days post injury (Hui et al., 2010). Functionally different macrophage subsets also exist in zebrafish (Nguyen‐Chi et al., 2015), the expression of a higher number of M2 type macrophage genes being upregulated very early after SCI whereas expression of only two M1 type macrophage genes with very low fold change was observed (Table 1) (Hui et al., 2014). So far all the circumstantial evidence puts forward a hypothesis that, in zebrafish SCI, there is an initial brief pro‐inflammatory state followed by an anti‐inflammatory response as M2 polarized macrophages persist and predominate. So there is a general bias towards an anti‐inflammatory state as M2 macrophages are not neurotoxic and hence lack of chronic inflammation promotes axonal regeneration after SCI. Further experimental analysis and functional validation is still required to fully uncover the role of different subsets of macrophages in controlling inflammation after SCI in zebrafish.

### 4.3 Inflammation after PNS injury—the role of macrophages and Schwann cells

Inflammation after PNS injury is linked to successful regeneration to some extent, and activation of Schwann cells and macrophages along with activation of a specific immune response are thought to be the underlying cause. Schwann cells express various inflammatory mediators such as tumor necrosis factor α (TNF‐α), IL‐1α and β, monocyte chemo‐attractant protein‐1 (MCP‐1), macrophage inflammatory protein 1, IL‐10, TGF‐β, and galectin‐3 in a specific temporal fashion. Schwann cells coordinate recruitment of activated macrophages (Gaudet, Popovich, & Ramer, 2011; Mietto, Mostacada, & Martinez, 2015) where inflammation is elicited through chemokines. A high fold increase of several pro‐inflammatory cytokines and chemokines is observed in PNS injury (Li et al., 2013). Analysis revealed that inflammation and immune response genes are in the top enriched categories and IL‐6 and Il‐10 pathways are the major signaling involved in nerve degeneration and regeneration. A comparative analysis of different chemokines and cytokines in mammalian and fish CNS and PNS after injury is listed in Table 1 (Knerlich‐Lukoschus & Held‐Feindt, 2015; Xing, Cheng, Zha, & Yi, 2017). Several cytokines, IL‐6, leukemia inhibitory factor (LIF) and ciliary neurotrophic factor (CNTF), are secreted from macrophages and Schwann cells and are upregulated in DRG neurons after PNS injury (Table 1) (Abe & Cavalli, 2008). Schwann cells not only are involved in recruitment of inflammatory cells but also terminate inflammatory response after PNS injury. Schwann cell remyelination of repairing axonal tract stimulates macrophage efflux from Schwann cell basal lamina via repulsive interactions between Nogo receptors (NgRs) in macrophages and ligands present in remyelinated axons (David, Fry, & López‐Vales, 2008).

The dynamics of macrophage polarization after PNS injury is still unclear, although it has been demonstrated that mouse PNS injury resulted in macrophage phagocytosis of myelin and stimulated M2 macrophage phenotype (Boven et al., 2006; Ydens et al., 2012). Infusion of IL‐4 cytokine into damaged sciatic nerve induced an M2 type macrophage response stimulating Schwann cell migration and improved axon regeneration of the distal nerve ends (Mokarram, Merchant, Mukhatyar, Patel, & Bellamkonda, 2012). Thus injury induced inflammation through various cytokines/chemokines triggers or enhances the fragmentation process. The activation of an immune response seems to be beneficial in peripheral nerve regeneration.

### 4.4 Some microglial responses after SCI in mammals and zebrafish and in PNS injury are common

There are some obvious similarities in the molecular signals emanating from macrophages and microglial cells that exist after SCI in mammals and fish. A recent study showed that blockage of lysophosphatidic acid, an important mediator of inflammation, improves outcome after SCI in both zebrafish and mouse models (Goldshmit et al., 2012). Several inflammatory stimuli such as lipopolysaccharide and zymosan can promote the M1 type macrophage phenotype and augment a regenerative response in both peripheral and central axons (Boivin et al., 2007; Gensel et al., 2009; Silver et al., 2015; Yin et al., 2003).

**Figure 2:**
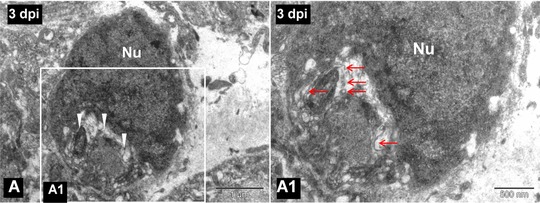
(A) TEM image of a 3‐day post injured spinal cord showing a phagocytic macrophage as cytoplasm is filled with myelin debris (white arrowheads). (A1) Higher magnification image of the boxed area in (A), in which degenerated myelin debris (red arrows) is clearly visible in the cytoplasm of the same phagocytic macrophage. Nu, cell nucleus. Scale bar 1 μm (A), 500 nm (A1)

Microglia are thought to be the first cell type reacting to CNS injury in both mouse (Bollaerts, Van Houcke, Andries, De Groef, & Moons, 2017; Czeh et al., 2011) and zebrafish (Baumgart, Barbosa, Bally‐cuif, Götz, & Ninkovic, 2012; Becker & Becker, 2001; Hui et al., 2010), although there may be temporal variation. The microglial response after injury in the CNS in mammals is mediated by ATP and receptor P2Y12 that serves as an attractant to reach the injury site (Davalos et al., 2005; Haynes et al., 2006). We confirmed the presence of activated microglia in early injured cord (Figure 2). In zebrafish also, ATP and P2Y12 purinergic receptor are required for microglial migration after CNS injury, which highlights a conserved role of these molecules by which microglia can sense neuronal damage (Sieger et al., 2012). Even in the absence of mechanical injury, when a single motor neuron was selectively ablated in zebrafish larval spinal cord, microglia became rapidly activated within 30 min as the dying neuron sent an “eat me” signal and subsequently became phagocytosed by microglia at the lesion site (Morsch et al., 2015). In PNS, ATP secretion from Schwann cell related lysosomal exocytosis during Wallerian degeneration has also been reported (Jung et al., 2014).

### 4.5 Zebrafish CNS injury exhibits a response similar to PNS injury

**Figure 3:**
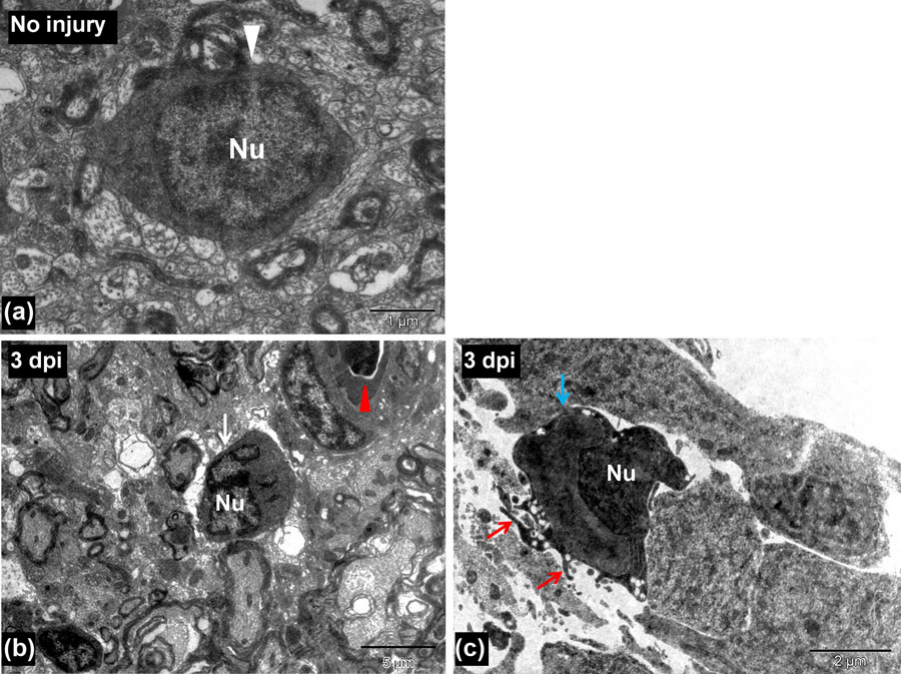
(A) TEM image of an uninjured spinal cord showing a microglia (white arrowhead). (B) TEM image of a 3‐day post injured spinal cord showing a microglia (white arrow) near the injury site. Red arrowhead indicates a blood vessel. (C) TEM image of a 3‐day post injured spinal cord showing an activated microglia near the central canal (having finger‐like cytoplasmic projections, red arrows) of injured spinal cord (blue arrow). Nu, cell nucleus. Scale bar 1 μm (A), 5 μm (B), 2 μm (C)

Previous investigations suggest that one of the reasons for failure of axonal regeneration after mammalian CNS injury is contributed by inefficient myelin clearance, whereas in PNS an efficient myelin clearance during Wallarian degeneration by Schwann cells and macrophages augments regeneration (David & Lacroix, 2003; Neumann et al., 2008; Vargas & Barees, 2007). In support of this concept, we observe that zebrafish macrophages are also involved in debris clearance, removal of apoptotic neurons, and engulfment of axonal fragments after SCI (Hui et al., 2010) (Figure 3). Following peripheral nerve injury in zebrafish, macrophages are recruited at the injured site long before axon fragmentation starts and is independent of Schwann cell derived signals. After axonal fragmentation, macrophages infiltrate towards the distal part of the injured nerve and engulf axonal debris (Rosenberg, Wolman, Franzini‐Armstrong, & Granato, 2012). Phagocytosis of debris following Wallarian degeneration is known to be pivotal for the successful repair of axons, and hence expression of the Wallarian degeneration slow (Wld^s^) protein delays or perturbs the axon regeneration process in both mouse and fish (Bisby & Chen, 1990; Chen & Bisby, 1993; Martin, O'Brien, Portera‐Cailliau, & Sagasti, 2010).

## 5 INTRINSIC CONTROL OF AXON GROWTH AFTER INJURY

A major limiting factor for successful axon regeneration of adult mammalian CNS is the poor intrinsic property of injured neurons. In contrast, following axotomy in PNS, a vast collection of regeneration associated genes (RAGs) are upregulated. Some directly control axonal regeneration whereas a few do not. c‐Jun (Raivich et al., 2004), ATF‐3 (Seijffers et al., 2006), Sox‐11 (Jankowski et al., 2009), SPRR1A (Bonilla et al., 2002), GAP‐43 and CAP‐23 (Bomze, Bulsara, Iskandar, Caroni, & Pate‐Skene, 2001) are responsible for neurite outgrowth. Activation of RAGs such as ATF‐3, c‐Jun, HSp 27, SPRR1A, GAP‐43, and the JAK‐STAT pathway occur in injured PNS axons, whereas injury to CNS axons does not lead to activation of these RAGs (Afshari et al., 2009; Mirsky & Jessen, 2012; Sun & He, 2010). In order to promote regeneration some effort has been made to induce the intrinsic growth capacity of adult CNS neurons, and some studies involving peripheral conditioning lesions showed that several regeneration associated transcription factors such as c‐Jun, CCAAT/enhancer binding protein, cAMP responsive element binding, STAT‐3, ATF‐3, SRY‐box 1 and Smad 1 can enhance growth of the central process beyond the lesion (Filous & Schwab, 2018). Gene profiling analysis led to identification of a number of genes involved in injured and regenerating neurons (Bosse, Küry, & Müller, 2001, 2006; Costigan et al., 2002; Küry et al., 2004; Michaelevski et al., 2010). These data identified 26 transcription factor families in PNS regeneration, including p53, Oct‐6, NF‐κB, NFATS, KLfS, Sox‐11, SnoN, ELK, P311, and E47 (Patodia & Raivich, 2012). Others (Chandran et al., 2016) used an in silico approach to identify “Hub” other factors or transcription factors associated with the RAG regulatory network in PNS which are not repeated in the CNS. They have also identified several novel RAGs in dorsal root ganglion neurons which need to be further validated in both PNS and CNS injury.

Several RAGs and growth associated proteins (GAPs) have been identified in fish after SCI or optic nerve injury, such as tubulin, cell adhesion molecules, microtubule interacting zRICH proteins, flotillins, and Reggie 1 and 2 (Rasmussen & Sagasti, 2017). Unlike mammals, regenerative responses in injured zebrafish CNS axons are high. The response could be elevated and sustained probably because of upregulation of several neuron intrinsic growth associated factors such as GAP‐43 and L1 related molecules and alpha tubulin (Becker et al., 2004; Kusik, Hammond, & Udvadia, 2010; Veldman et al., 2010). Axonal regeneration in both zebrafish CNS and PNS decline with age (Becker et al., 1997, 1998; Graciarena, Dambly‐Chaudière, & Ghysen, 2014) and low regenerative capacity of these axons is often related to a failure to upregulate GAPs (Rasmussen & Sagasti, 2017). Some of the RAGs identified in fish are also capable of axonal regeneration in mammals. Furthermore, our array analysis data suggest that several RAGs associated with regeneration of PNS are upregulated after SCI in zebrafish (Hui et al., 2014). Specific intrinsic molecular differences contribute to differential axonal growth response in PNS and CNS injury, and exploitation or manipulation of specific PNS related RAGs could promote limited CNS regrowth after injury. Thus one of the future strategies to induce regeneration in mammalian CNS would be manipulation of RAGs following injury.

CNS axons are capable of regenerating over long distances within a permissive environment (David & Aguayo, 1981) and several factors in CNS myelin contribute to regeneration failure in mammals. However, it was observed that elevation of intracellular cAMP could effectively rescue the myelin inhibitory effect. A high level of cAMP is responsible for the conditioning lesion effect observed following PNS injury and can promote axonal growth in the presence of myelin. cAMP upregulates several genes like arginase I and IL‐6. cAMP analogs can also improve CNS axon regeneration in goldfish and cAMP induced regeneration of zebrafish (Bhatt et al., 2004).

It has been reported that either suppression of inhibitory factors such as phosphatase and tensin homolog (PTEN), suppressor of cytokine signaling (Socs3) or overexpression of genes involved in PNS regeneration such as Klf7, CREB, and c‐Jun could induce axonal regrowth in adult CNS (Blackmore et al., 2012; Gao et al., 2004; Lerch, Martínez‐Ondaro, Bixby, & Lemmon, 2014; Smith et al., 2009, 2011; Sun & He, 2010). Several negative regulators of intrinsic axonal growth such as cAMP, PTEN and Socs3 in mammals also play a conserved role in zebrafish (Diekmann, Kalbhen, & Fischer, 2015; Elsaeidi et al., 2014; Liu, Tedeschi, Park, & He, 2011, 2014; Smith et al., 2009). PTEN inhibition in cortical neurons of mice showed robust regrowth of injured corticospinal tract axons which are otherwise refractory to regeneration (Liu et al., 2010; Lu, Belin, & He, 2014). Similarly mammalian target of rapamycin (mTOR) activation promotes axon regeneration. Activating PI3K or inhibition of PTEN in DRG neurons increases neurite outgrowth. Blocking mTOR by rapamycin fails to inhibit neurite growth of DRG neurons, suggesting that an mTOR independent mechanism may mediate the regeneration of peripheral sensory axons. It is apparent that different mechanisms regulate the effects of the PTEN/PI3K pathway in the axonal regeneration of PNS and CNS (Lu et al., 2014; Ohtake et al., 2015). To understand whether active mTOR plays a role in zebrafish optic nerve regeneration, Diekmann et al. (2015) studied mTOR signaling after injury and concluded that regulation of mTOR activity after optic nerve injury is different from that of mammals and plays a supplementary role. In zebrafish SCI, morpholino mediated Ptena inhibition improved axonal regrowth from the nucleus of medial longitudinal fasciculus (NFML) in the brainstem (Liu, et al., 2014). We reported upregulation of both Stat3 and Socs3 after SCI in fish and observed axonal regrowth (Hui et al., 2014). The specific functional role of Stat3 and Socs3 in zebrafish CNS axons remains to be elucidated further. Stat3 has been implicated in both PNS and CNS regeneration although expression is significantly higher in PNS rather than CNS neurons. Stat3 exhibits phase‐specific regulation of axonal regeneration after mammalian CNS and PNS injury (Bareyre et al., 2011). Furthermore overexpression of hyperactivated Stat3 can also promote neurite outgrowth in optic nerve injury (Mehta et al., 2016).

## 6 EXTRINSIC FACTORS—CNS MYELIN ASSOCIATED INHIBITORS AND THEIR ROLE IN AXONAL REGROWTH

Following injury, the PNS axons in higher vertebrates are capable of regeneration whereas CNS axons fail to regenerate. This difference in regeneration capacity is not only because of differential intrinsic properties of CNS and PNS neurons but also due to the respective environments. The extrinsic factors include extracellular matrix (ECM), trophic factors, chemorepulsive guidance cues, and myelin associated lipids and proteins. Accumulation of myelin breakdown products released from severed axons and the formation of inhibitory glial scar at the injury epicenter lead to a chemical and physical barrier that perturbs axonal growth and regeneration (Busch & Silver, 2007; Fawcett, 2006; Schwab, 2004). The major extrinsic barriers to axonal regrowth in injured CNS are several growth inhibitory and repulsive factors expressed by different glial cells such as glial progenitors, oligodendrocytes, astrocytes, and microglia (Silver et al., 2015). Regenerating axons of both fish and mammalian neurons are prevented by mammalian oligodendrocytes and myelin, although axons can grow in the presence of fish oligodendrocytes (Bandtlow, Zachleder, & Schwab, 1990; Bastmeyer, Beckmann, Schwab, & Stuermer, 1991; Fawcett, Rokos, & Bakst, 1989; Schwartz et al., 1985; Wanner et al., 1995). This experimental evidence indicates that the factor(s) in the environment of mammalian CNS and PNS may account for a differential axonal regeneration response (David & Aguayo, 1981). Distinct factors in the mammalian and fish CNS environment account for regeneration impermissive and permissive niches respectively. Unlike mammals, zebrafish oligodendrocytes upregulate several recognition molecules such as contactins, Po, and L1 related molecules (Becker & Becker, 2014) which promote axonal regeneration.

### 6.1 Adult CNS myelin is inhibitory for neurite outgrowth in mammals

CNS myelin is found to be the primary source of inhibition as immobilized CNS myelin, but not PNS myelin, is the one that inhibits axonal outgrowth (Schwab & Thoenen, 1985). The three isoforms of Nogo (Nogo A, B, and C) belonging to the reticulon family (RTN 4) of membrane proteins are present in mammalian CNS. Among these, Nogo A is highly expressed in oligodendrocytes in mammalian CNS after injury (Huber, Weinmann, Brösamle, Oertle, & Schwab, 2002). Both in vitro and in vivo experiments suggest that the application of anti‐Nogo antibodies causes axonal sprouting in injured adult mammalian CNS (Fouad, Klusman, & Schwab, 2004; Freund et al., 2006; Schnell & Schwab, 1990). Both the amino terminal and Nogo‐66 domain are inhibitory to neurite outgrowth (Fournier, GrandPre, & Strittmatter, 2001; Oertle et al., 2003; Prinjha et al., 2000).

Other CNS myelin associated factors that can strongly inhibit axon outgrowth in vitro include myelin associated glycoprotein (MAG) (McKerracher et al., 1994; Mukhopadhyay et al., 1994), oligodendrocyte myelin glycoprotein (OMgp) (Wang et al., 2002), semaphorins 4D/CD100 (Moreau‐Fauvarque et al., 2003) and 5A (Goldberg et al., 2004), ephrin B3 (Benson et al., 2005) and A5, as well as CSPG and myelin glycolipid sulfatide (Yiu & He, 2006). All these agents have a growth inhibitory effect on a variety of neuronal cells in vitro (Fawcett, Schwab, Montani, Brazda, & Müller, 2012; Giger, Hollis, & Tuszynski, 2010; Sandvig, Berry, Barrett, Butt, & Logan, 2004). Although there are diverse components in myelin exerting an inhibitory effect the respective contributions of individual factors are not well understood. It seems that the inhibitory effect of CNS myelin is exerted by a significant degree of overlap and cross‐regulation amongst these factors.

### 6.2 Involvement of multiple ligands and multiple receptors in axonal growth inhibition

Amongst several ligands MAG is synthesized in oligodendrocytes and Schwann cells, but it is a relatively minor constituent of both CNS and PNS myelin (Quarles, 2007). Although MAG is responsible for maintenance of myelinated axons, it has been widely used as an inhibitory substrate for neurite outgrowth assay in postnatal and adult neurons. It serves dual functions depending on age and type of neuron, i.e., promotes neurite outgrowth in young and inhibits the same in adult neurons. Furthermore, genetic deletion studies revealed that MAG may have opposing roles—inhibitory on some neurons (reduced sprouting of corticospinal tract axons) while promoting on others (enhanced serotonergic axon sprouting) even in adult CNS. All the genetic deletion analysis as well as in vitro and in vivo experimental evidence suggests that MAG may take a divergent role in disease and injury. It may promote axonal growth and protect axons from further degeneration, contrary to its well publicized role in axonal growth inhibition.

OMgp, a glycosyl phosphatidyl inositol (GPI) linked protein containing a leucine rich repeat (LRR) domain, is widely distributed in the CNS and causes growth cone collapse in many neuronal populations. It has also been demonstrated that this protein is enriched in the membranes of oligodendrocyte‐like cells around the nodes of Ranvier, hence damaging collateral sprouting (Huang et al., 2005).

Generation of dominant negative NgR mutation and antibody to NgR suggests that both are capable of blocking neurite outgrowth by MAG (Domeniconi et al., 2002). Major myelin inhibitors like Nogo, MAG, and OMgp all inhibit neurite outgrowth through engagement of NgR and the inhibitory effect is mediated by activation of the small GTPase signal transducer Rho, followed by ROCK and actin regulator slingshot and cofilin. The most important player of Nogo inhibition is the Nogo‐66 domain, which interacts with NgR, a GPI linked receptor protein. Both p75 (NGFR) and LINGO‐1 have been identified as co‐receptors for NgR. Recently identified Nogo A specific receptor sphingosine‐1‐phosphate receptor 2 (9S1PR2), several Eph receptors, semaphorin receptors, and CSPG‐interacting proteins LAR and protein tyrosine phosphatase (PTPase) σ are all implicated in growth cone collapse and growth inhibition of their corresponding ligands. So far, many axonal inhibitory molecules have been identified, which prompted the discovery of the corresponding receptors. The identification and use of such receptor−ligand complexes could pave the way to understanding the major impediment to regrowth in injured CNS and to targeting specific receptor−ligand complexes for potential therapeutic strategies.

Myelin associated inhibitory molecules like Nogo and MAG are expressed in zebrafish CNS (Hui et al., 2014; Lehmann et al., 2004; Pinzon‐Olejua, Welte, Abdesselem, Malaga‐Trillo, & Stuermer, 2014; Shypitsyna, Málaga‐Trillo, Reuter, & Stuermer, 2010). It has been reported that the mammalian Nogo‐A‐specific region is absent in zebrafish (Shypitsyna et al., 2010); hence RTn4a does not play an axon inhibitory role after injury (Abdesselem et al., 2009). The receptors such as NgR (receptor for both Nogo‐66 and CSPG), RPTP sigma and LAR are also present in zebrafish CNS (Abdesselem et al., 2009).

## 7 GLIAL SCAR FORMATION AFTER MAMMALIAN SCI

Apart from degenerating myelin, astrocytic scar formation has been regarded as an important component of axonal growth inhibition after injury in the mammalian CNS. The glial scar is a complex of ECM and cell types that form a dense structure, which may serve to protect adjacent tissue but may also be a major impediment to regenerating axons. Various previous reports correlate the failure of axonal regrowth to the presence of mature astrocytes, astrocytic scars, and CSPG produced by astrocytes (Liuzzi & Lasek, 1987; Silver & Miller, 2004). However, recently Anderson et al. (2016) challenged the dogma that the glial scar is the major impediment to axonal regrowth and functional recovery. The astrocyte response to injury is termed reactive gliosis which is characterized by cellular hypertrophy, changes in gene expression, and cellular proliferation (Sofroniew, 2005, 2009). While studying the molecular mechanism underlying reactive gliosis and its effect on astrocyte function, it has been suggested that astrogliosis may result in both beneficial and detrimental effects on axonal growth depending on its time course and dynamic features (Bush et al., 1999; Faulkner et al., 2004; Wanner et al., 2013). Some of these responses may have beneficial properties causing reduction of inflammation and cellular degeneration, while a particular population of astrocytes may support axonal regrowth (Faulkner et al., 2004). The astrocytic scar or glial scar is made up of two distinct components: (a) the lesion penumbra with hypertrophic astrocytes and (b) the core of the lesion composed of NG2^+^ oligodendrocyte precursors, PGDFRβ^+^ fibroblasts, and activated infiltrating macrophages and pericytes. Dystrophic axons within the lesion remain in close vicinity and make stable contact to NG2^+^ glia (Busch et al., 2010; McTigue, Tripathi, & Wei, 2006). Thus the presence of segregated populations of different cells within structurally layered glial scars is indicative of the existence of specific chemorepulsive/attractive mechanisms (Cregg et al., 2014). NG2^+^ glia initially provide a supportive substance which helps to prevent axonal dieback due to the presence of inflammatory cells. Later, the interaction causes entanglement of dystrophic axons to NG2 glia. These synaptic specializations of dystrophic axons and NG2^+^ glia are responsible for hindering axonal growth. Although OPC proliferation is inhibited by NG2, its protein scaffold can also be degraded by MMP‐9, which in turn can facilitate remyelination.

### 7.1 CSPG mediated growth inhibition

Astrocytes are considered to be the major cell type in glial scars and produce different classes of proteoglycans, such as heparin sulfate proteoglycan, dermatan sulfate proteoglycan, keratin sulfate proteoglycan, and CSPG (Johnson‐Green et al., 1991; Silver & Miller, 2004). Expression of CSPG increased markedly after CNS injury, in the vicinity of a disrupted blood−brain barrier. Evidence suggests that the inhibitory activity of CSPGs depends on the GAG components as these studies demonstrated that treatment with chondroitinase ABC removes sulfated GAG chain from CSPG and hence abolishes inhibition (Carulli, Laabs, Geller, & Fawcett, 2005; Silver & Miller, 2004). Chondroitinase treatment not only enhances axonal regeneration and functional recovery after SCI, but promotes collateral sprouting and generation of new synapses (Alilain, Horn, Hu, Dick, & Silver, 2011; Bradbury et al., 2002). Although it is clear that CNS myelin and glial scars both inhibit axonal regeneration, their relative importance in vivo is not very convincing as there are conflicting reports suggesting that some overlap and spatiotemporal difference exists in regulation.

Several general mechanisms are implicated for CSPG‐mediated growth inhibition, such as masking of neuronal integrin interaction with growth promoting ECM, e.g., laminin, NCAM, etc., facilitation of inhibitory effects of Sema5a, and limiting calcium availability to neurons by binding extracellular Ca^+^ and by binding with CSPG receptors. Several CSPG receptors like LAR phosphatase, PTPσ, Ngr1, and Ngr3 (Lutz & Barres, 2014) have been reported. The intracellular consequences of CSPG mediated growth inhibition involve Rho activation, AKT inactivation, and calcium related signals like PKC (Yiu & He, 2006). Interestingly, the presence of both the growth promoting and the growth inhibitory components in the ECM and CSPG might act by interacting with growth promoting substrates. Conversely, exposure of CSPG can also enhance dystrophic growth cone formation in injured sensory axons, suggesting that CSPG mediated inhibition could affect both cytoskeletal and membrane elements of the growth cone structure (Ramon & Cajal, 1928; Tom et al., 2004).

## 8 ABSENCE OF ASTROGLIOSIS IN REGENERATING ADULT ZEBRAFISH CNS

In adult zebrafish, the most important aspect of axonal regeneration is that the regenerated axons reach the appropriate targets over long distances and can make re‐innervations, a phenomenon that does not happen in the case of mammals. It has been hypothesized that regenerating axons could trace their original pathways along the degenerating tracts as happens in PNS (Graciarena et al., 2014). It has been proposed that regenerating axons re‐route through gray matter during regeneration which does not match the previous hypothesis (Becker & Becker, 2001). So what would be the guidance cues in adult regenerating axons in zebrafish? CSPG and other inhibitory molecules are known to play an important role during development by repelling axons from certain areas that are not meant to be innervated. Degradation of CSPG by intra‐ventricular injections of chondroitinase showed that regenerating zebrafish optic axons are indeed repelled by CSPGs in the posterior pretectal nucleus (Becker & Becker, 2002).

The formation of a glial scar as discussed earlier proved to be a major impediment to mammalian axonal regrowth. Astrocytes and NG^+^ oligodendrocyte progenitors are at the center of reactive gliosis. Attenuated reactive astrogliosis in GFAP^−/−^ vimentin^−/−^ mice show reduction of glial scar formation (Pekny & Pekna, 2014). Unlike mammals, in adult zebrafish CNS we observed downregulation of some of the markers such as GFAP and vimentin immediately after injury. Furthermore, the glial composition and response in fish may vary from mammals. The presence of radial glia rather than parenchymal astrocytes and a different glial response after injury, i.e., the presence of microglia and macrophages and the absence of GFAP^+^ radial glia and adult oligodendrocytes near the injury site, indicate the generation of a different glial environment and absence of a glial scar. Furthermore, while identifying the different proliferating progenitors we observed a high number of proliferating radial glia and a limited number of NG2^+^ oligodendrocyte progenitors in regenerating cord (Hui et al., 2015). There is an obvious lack of a permanent glial scar and formation of a growth permissive glial bridge that may lead to augmentation of axonal regeneration in adult zebrafish CNS (Baumgart et al., 2012; Goldshmit et al., 2012; Hui et al., 2010). Strand et al. (2016) reported increased β‐catenin signaling after SCI in zebrafish. Overexpression of Dkk 1b inhibits Wnt β‐catenin signaling and functional recovery. β‐catenin signaling is necessary for glial bridge formation and Dkk 1b overexpression inhibits axonal regeneration. After SCI in zebrafish larva, radial glia the known precursors for generating neuron exhibit wnt/b catenin signaling and exposing larval zebrafish to IWR1 inhibits Wnt/b catenin signaling preventing axonal elongation in the injury epicenter (Briona, Poulain, Mosimann, & Dorsky, 2015). Absence of CSPG and gliosis following SCI has also been reported in another teleost model *Apternotus leptorhynchus* (Vitalo, Sîrbulescu, Ilieş, & Zupanc, 2016). Application of Fibroblast Growth Factor‐2 (FGF2) after mammalian SCI generates a radial‐glia‐like or pro‐regenerative glial‐progenitor‐like state and thus FGF2 mediated response leads to a change in glial morphogenesis and attenuated scar formation in both mammalian and zebrafish SCI (Goldshmit et al., 2014).

The composition of CNS ECM is different from the PNS. The dense glial scar and ECM network act as a physical and molecular barrier to axon regeneration in injured CNS. The major ECM molecules in the CNS include a huge amount of glycosaminoglycan hyaluronan and the glycoproteins tenascin‐C and thrombospondin whereas PNS ECM includes laminins, collagen, and heparin sulfate proteoglycan. The mechanism of glial bridge formation seems to be conserved between fish and mammals, since application of FGF2 improves recovery after mammalian SCI and promotes bridge formation in zebrafish (Goldshmit et al., 2014). The glial bridging behavior also resembles Schwann cell bridging in mammalian PNS. Schwann cells secrete a basal lamina rich in growth promoting ECM, which is crucial to the ability of these cells to myelinate (Bunge et al., 1990). Upregulation of pro‐regenerative ECM molecules like laminin−integrin interaction could trigger PI3K activation, Akt signaling, and cytoskeletal rearrangements, all these signals play important role in PNS regeneration (Chen, Yu, & Strickland, 2007), although in intact peripheral nerve Schwann cells express CSPG and after injury CSPG expression is upregulated (Höke et al., 2006). Basal lamina tubes play a vital role in shielding axons from CSPGs inside endonurium. Furthermore, upregulation of MMP‐2 and MMP‐9 in the distal stump of injured PNS relieves CSPG inhibition by degrading the same and favoring a more regeneration permissive environment for axonal growth (Ferguson & Muir, 2000). Similar to Schwann cells, astrocytes may upregulate ECM molecules like fibronectin and laminin after injury, but modest upregulation of pro‐regenerative CNS ECM is overshadowed by a huge upregulation of CSPGs which are inhibitory to axonal regrowth (McKeon, Höke, & Silver, 1991, 1995) in CNS.

After injury in the CNS, a complex ECM environment is generated in the wound site that may be inhibitory for axon regrowth. The CNS glia and ECM are known to contribute to inhibitory scars whereas PNS glia affect post injury ECM favoring axon growth. In zebrafish spinal cord, Wnt signaling is activated after SCI that controls fibroblast and ECM deposition. Wehner et al. (2017) demonstrated that Wnt/b catenin signaling controls collagen XII deposition and promotes axonal regeneration after SCI. Similarly, a fibrous scar was generated after SCI in goldfish but, despite the fibrous scar, regenerating axons can enter and pass the lesion aided by the glial process (Takeda, Atobe, Kadota, Goris, & Funakoshi, 2015).

## 9 DEMYELINATION AND REMYELINATION AFTER INJURY

During CNS injury axons are severed and cells in the white matter may die. Demyelination of axons following injury results in severe loss of function and has grave consequences in both the CNS and PNS. So, remyelination proves to be a crucial step to achieve successful axonal regeneration followed by appropriate target innervation and functional recovery. NG2^+^ glia play a prime role in demyelination and remyelination in adult mammalian CNS. These cells proliferate and produce a large number of new oligodendrocytes (Ishii et al., 2001). A few critical ingredients of remyelination include differentiation of progenitors either to oligodendrocytes or Schwann cells, trophic factors, and environmental signals that govern myelination/remyelination and removal of myelin debris. The efficient removal of myelin debris facilitates differentiation of progenitors and permits successful remyelination of damaged axons. As functional regeneration can happen both in the PNS and CNS of fish and urodele amphibians, axonal regeneration has been studied in both these scenarios in fish. While studying the myelination and remyelination process in the zebrafish nervous system it has been observed that the fundamental structure and composition of myelin and the underlying molecular mechanism controlling myelination are conserved between fish and mammals (Preston & Macklin, 2015). OPCs generate myelin forming oligodendrocytes continuously in adult rodent brain (Rivers et al., 2008). Similarly, the number of oligodendrocyte lineage cells increases and axon myelination occurs continuously in post embryonic fish cord by Myelin Basic Protein (MBP) oligodendrocytes (Jung et al., 2010; Park, Shin, Roberts, & Appel, 2007). Unlike mammalian CNS myelin, fish myelin and oligodendrocytes do not inhibit axon regrowth (Bastmeyer et al., 1991). Following CNS injury in zebrafish, the expression of growth promoting cell surface protein is linked to axonal regeneration. Myelin protein zero in mammalian Schwann cells is known to promote axonal growth whereas *mpz* genes encoding zebrafish ortholog Po and contactin 1a are strongly upregulated in oligodendrocytes in regenerating white matter tracts after injury (Schweitzer et al., 2003; Schweitzer et al., 2007). It has also been observed that separate *cis*‐regulatory elements mediate *mpz* expression in myelinating oligodendrocytes and its transcriptional induction by axonal damage. Thus different mechanisms exist during myelination and maintenance of CNS myelin in uninjured and injured zebrafish CNS (Bai, Parris, & Burton, 2014). Interestingly the anatomical characteristic of zebrafish cord refers to the fact that axons of ventral and dorsolateral cord are highly myelinated, whereas other regions of the white matter harbor branches of radial glia along with non‐myelinated axons. This may indicate that unmyelinated axon bundles and radial processes may provide a more permissive cellular environment for axonal regeneration after injury in adult zebrafish cord.

**Figure 4:**
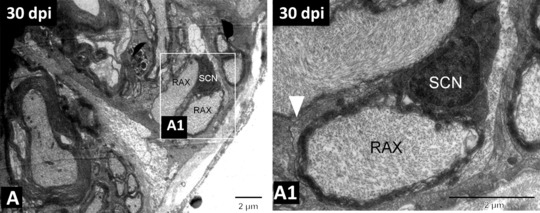
(A) TEM image of 30‐day regenerated spinal cord showing remyelinating axons wrapped by a Schwann cell. (A1) Higher magnification image of the boxed area in (A), in which the white arrowhead indicates the basal membrane of the Schwann cell, remyelinating an axon. SCN, nucleus of the Schwann cell; RAX, regenerating axon. Scale bar 2 μm (A), (A1). Adapted from Hui et al., 2010

The regenerative capacity of axons is dependent on and supported by Schwann cells—myelin forming glias of the PNS. As studied in a variety of paradigms, injury to the PNS would cause Wallarian degeneration, followed by neurite regeneration and remyelination (Chen et al., 2007; Scheib & Höke, 2013). During Wallerian degeneration, Schwann cells dedifferentiate and participate in phagocytosis of their own myelin sheath and recruit macrophages, which are critical for removal of damaged tissue. Several signaling pathways like JNK/c‐Jun, ERK, Notch, and p38 are responsible for Schwann cell injury response. After successful axonal regeneration Schwann cells remyelinate by producing a significantly thinner myelin sheath (Glen & Talbot, 2013). Several trophic factors like neurotrophins, neurogulin 1/ErbB signal, the ADAM secretase family, small molecules like apolipoprotein E, ascorbic acid, etc. play a critical role in the maintenance and repair of the PNS (Taveggia, 2016; Zhou & Notterpek, 2016). Conditional knockout of NgR1‐I in Schwann cells results in severe defects in remyelination. The peripheral axons of sensory neurons regenerate quickly after injury in zebrafish (Graciarena et al., 2014). Upon denervation, Schwann cells downregulate the expression of P_0_‐like myelin protein and claudin‐K and facilitate axonal regrowth (Xiao et al., 2015). Xiao et al. showed that Schwann cells facilitate but are dispensable for axonal regrowth although these cells are necessary for the re‐innervation of peripheral targets. The behavior of Schwann cells is similar in mammals and fish in promoting axonal growth. During Wallerian degeneration transdifferentiated Schwann cells myelinate, redifferentiate upon entering into contact with regrowing axons, and represent a unique population of repair cells designated for regeneration of the PNS (Arthur‐Farraj et al., 2012; Xiao et al., 2015). CNS remyelination is usually mediated by oligodendrocytes but it can also be mediated by Schwann cells. The cellular origins of remyelinating oligodendrocytes and Schwann cells in the CNS have not been resolved. Remyelinating Schwann cells within the CNS are generally believed to migrate from a PNS source such as spinal or cranial roots. A recent genetic fate mapping study in mice showed that DGFRα/Olig expressing precursors give rise to all remyelinating oligodendrocytes. Furthermore, the majority of remyelinating Schwann cells within the CNS are generated from OPCs, not from Po expressing Schwann cells (Zawadzska et al., 2010). After zebrafish SCI, we observed axonal regrowth and functional recovery. We observed debris clearing by macrophages followed by the presence of Schwann cell progenitors around the remyelinating axons (Figure 4) (Hui et al., 2010), although we cannot confirm the origin of these remyelinating cells. It is imperative to identify the extent to which either CNS precursors or PNS Schwann cells contribute to remyelination after SCI in fish. This would have serious implications in developing future strategies for remyelination and inducing successful axonal regeneration after SCI.

9.1

#### 9.1 Axonal guidance molecules

The precise axonal connections and wiring to appropriate targets in adult CNS occur during development and the immediate perinatal period. When adult mammalian CNS is injured this intricate system fails to regenerate. Interestingly, many guidance and synaptogenic cues that regulate neural wiring during CNS development also contribute to the remodeling of axonal connections in injured spinal cord. Apart from several extracellular matrices, many guidance molecules like the netrins, semaphorins, the robo/slit family, ephrins, Wnts, and repulsive guidance molecules have been identified and are known to play a role after SCI in mammals (Giger et al., 2010; Harel & Strittmatter, 2006; Hollis, 2016; Jacobi, Schmalz, & Bareyre, 2014). It has been suggested that inhibitory and chemo‐repulsive axon guidance molecules are likely to play an important role in synaptic stabilization and limitation of neuronal plasticity in adult life. Significantly, after SCI, several members of the slit and semaphorin family, syn‐CAM, neuroligin, and ephrin B are expressed in spinal neurons, for e.g., slit 1‐3 and semaphorin 7a and neuroligin‐1 are expressed in propriospinal neurons and a few glycinergic interneurons in spinal cord (Jacobi et al., 2014). Wnt signaling in adult CNS injury also contributes to brain and spinal cord circuitry (Onishi et al., 2014). The reappearance of developmental cues after injury limits both descending and ascending motor and sensory axons. In particular Wnt‐PCP signaling plays an important role in axon guidance. Ryk mediates Wnt repulsion by inhibiting PCP signaling. The role of Wnt in directing peripheral axon growth has not been studied in its entire gamut. But Schwann cells in specific regions express glycosyl transferase Ih3 resulting in collagen4a5 expression and thus repel axons towards inappropriate trajectories (Isaacman‐Beck et al., 2015). Several axonal attractants/repellants are upregulated in zebrafish SCI (Table 1) (Hui et al., 2014). Regenerating zebrafish optic nerve axons are sensitive to axonal guidance molecules like CSPG and tenascin‐R (Becker & Becker, 2002). Moreover, optic tectum expresses axon repellant ephrin‐A2 and ephrin‐A5b (Becker & Becker, 2000). Several motor guidance and target recognition molecules such as CAMs, semaphorins, netrins, robo/slit, ephrin and rtk are also expressed in the developing nervous system.

#### 9.2 Future perspectives

The similarities and differences of molecular and cellular mechanisms of adult zebrafish CNS regeneration and mammalian PNS regeneration give us insights to better understand how a permissive niche can be created to achieve successful axonal regeneration in the mammalian CNS and to adopt successful therapeutic strategies. The abundant data discussed above provide a strong basis to pursue research on zebrafish, with a goal to induce regeneration in mammals including humans. To consider some of the future therapeutic strategies a better understanding of the differential immune response after injury is required. Zebrafish could serve as an ideal model to uncover the beneficial immune response and maintenance mechanism for successful regeneration. To induce axon regeneration, understanding glia present in the axon environment and their response to injury is crucial. A deeper understanding of the mechanism employed by PNS glia could shed more light on the regeneration permissive environment. Successful axonal regrowth in zebrafish CNS relies on high intrinsic capacity, absence of a glial scar, and appropriate axon guidance molecules. Future studies can be facilitated by large scale gene expression analysis to identify novel RAGs, axonal guidance molecules, etc. along with efficient functional analysis by adapting reverse genetic techniques paving the road for rapid identification of the molecular pathways involved in axonal regeneration. A continued focus on comprehensive dissection of the molecular mechanisms of endogenous capacity of axonal regeneration in zebrafish could be maximally exploited to achieve functional recovery after CNS injury.

A significant focus of SCI research has been directed towards the mammalian model, which is regeneration incompetent. There has been much progress to understanding the cellular and molecular basis of regeneration failure in mammalian CNS, yet successful repair strategies cannot be adopted from mammalian SCI. The injury response in mammals proves to be complex and dynamic. The failure and success of regeneration depend on several cellular events and are controlled by many event‐specific molecules and factors. However, much of the therapeutic strategy to induce regeneration, particularly in spinal cord, is actually based on specific molecules which may have a role in controlling any specific events. In order to improve therapeutic intervention after SCI, a combinatorial approach should be taken. Manipulation of a single event, molecule, or a group of molecules may not improve the clinical outcome. Serious thought has to be given to a more comprehensive approach. In such a scenario a translational approach by studying a model organism to enhance CNS regeneration is invaluable and needs further attention.

## CONFLICT OF INTEREST

No conflict of interest.
